# Editorial: Recent advances and challenges in electron microscopy characterizations of radiation-sensitive nanoparticles

**DOI:** 10.3389/fchem.2023.1171240

**Published:** 2023-03-02

**Authors:** Wai Li Ling, Yuki Kimura, Yu Han, Yanbin Li

**Affiliations:** ^1^ Université Grenoble Alpes, CEA, CNRS, IBS, Grenoble, France; ^2^ Hokkaido University, Sapporo, Japan; ^3^ King Abdullah University of Science and Technology, Thuwal, Saudi Arabia; ^4^ Stanford University, Stanford, CA, United States

**Keywords:** transmission electron microcopy (TEM), artificial intelligence, radiation sensitivity, carbohydrate, electron tomography (ET), perovskite, ice phase transition, liquid cell electron microscopy

Electron microscopy has welcomed many important advancements in the last decade. The availability of direct electron detectors and new image processing algorithms led to a breakthrough in protein structure resolution, which had historically been limited by radiation damage in protein molecules (Kuhlbrandt, 2014). Since then, detectors, data acquisition strategies, and image analysis algorithms have continued to evolve and improve. For instance, the new generation of hybrid-pixel detectors not only boasts single-electron sensitivity but also high speed as well as high dynamic range. Algorithms using artificial intelligence (AI) or machine learning are applied more and more often in different stages of electron microscopy experiments, overcoming certain human limitations. These new advancements have benefited not only biological samples, but also other radiation sensitive materials that could not have been studied by electron microscopy before.

This Research Topic presents two reviews and three original research articles addressing the Research Topic of electron microscopy on radiation sensitive samples from diverse aspects.

The review by Xue et al. summarizes the different mechanisms of radiation damage by high-energy electrons and discusses the experimental parameters to consider for limiting radiation damage in cryo-electron tomography. Tomography reconstructs the 3-dimensional (3D) structure of an individual object (non-averaging) using a set of its projections from different angles. As such, the technique is highly dose-intensive as the sample is subjected to many exposures to the electron beam as projections at different angles are acquired. The reward is that 3D structures of unique objects can be reconstructed. Indeed, with the data Research Topic strategies and image analysis algorithms developed in the Ren group and described in the review, 3D structures of individual particles of radiation-sensitive biomaterials have been reconstructed with sub-nanometer resolution (Zhang and Ren, 2012; Lei et al., 2018).

The mini-review by Ogawa and Putaux highlights electron microscopy on carbohydrate nanoparticles, which are ubiquitous and versatile but have little structural information to date. For example, cellulose forms structural building blocks whereas starch granules make efficient fuel storage. Yet, little is known of their structural organization or the effects of additives or defects. Structural studies, which have been limited by the radiation sensitivity of these organic particles, can reveal the local structure that determines their physical properties. As carbohydrate nanoparticles are non-toxic, biodegradable, and renewable, they are very attractive for a wide range of applications, from medicine to food packaging (Feng et al., 2021). Recent advances in electron microscopy will surely accelerate the understanding of carbohydrate nanoparticle structure and the development of their applications.

The research article by Duong et al. examines the practice of high-resolution electron microscopy on CsPbBr3 nanoparticles. These perovskite nanocrystals are inorganic but their highly ionic nature renders them radiation sensitive. They have exceptional optical and electronic properties and have great potential in many applications, including light-emitting devices and photoreduction. Their structural studies had been controversial due to their susceptibility to radiation damage. Duong et al. demonstrate that the native structure of these nanocrystals can be revealed by direct imaging or diffraction by combining the use of a hybrid-pixel detector, low-temperature imaging, low-dose image acquisition, and graphene sample support film. Lessons learned from these studies can evidently be applied to electron microscopy studies of other radiation-sensitive materials.

Diverging from the above works, which orient towards materials with technological applications, Kouchi et al. investigate a rare phase of ice important to planetary science. The formation of this ice phase is sensitive to the type as well as the intensity of the radiation. To investigate the transition to this ice phase, Kouchi and others describe the instrumentation necessary for the studies, including an ultrahigh vacuum microscope and ports for the deposition of ice as well as for the controlled ultraviolet irradiation. A special liquid-He cooled holder was also used. Besides using techniques to ensure that the imaging electron beam does not interfere with the experiment of the radiation-sensitive ice-phase transition, the article also describes techniques for measuring the ice thickness. Readers will find rich information on how an electron microscope can be tailored for the studies of special samples.

From space science, we get to the most futuristic article in this Research Topic, which describes AI-assisted experiment monitoring and data Research Topic. Katsuno et al. use machine learning to detect nucleation events. Nucleation is stochastic and determines the crystallinity and thus the physical properties of the resulting crystal. Nucleation is important in all branches of science and technological developments. The classical nucleation theory has been the general understanding but has been challenged in recent years (Van Driessche et al., 2021). To study nucleation, cryo-electron microscopy has been used to capture snapshots of the events (Van Driessche et al., 2022). Although this method can give important insights, success is not assured in each experiment. Nuclei are not necessarily captured due to the stochastic nature of nucleation and also due to the sample reduction during the vitrification process. In their experiments, instead of contending to mitigate radiation damage, Katsuno et al. exploit the energy from the electron beam to induce nucleation. The nucleation events are then detected by the early detection system trained by machine learning. The tools developed in this study will definitely benefit the study of nucleation and other dynamical events in liquid-cell electron microscopy.

Though our Research Topic comprises a small number of articles, these articles span a wide range of samples and perspectives on electron microscopy and radiation sensitive materials. The nice assortment testifies to the interdisciplinarity of the Research Topic. As technology involves more and more organic and biomaterials, we hope that the information in this Research Topic can be applied to diverse disciplines and contribute to the advancement of various fields of science.
